# The Impact of the COVID-19 Pandemic on Self-Harm Attempts Observed in a Hospital Emergency Department

**DOI:** 10.3390/healthcare12030385

**Published:** 2024-02-02

**Authors:** Elena Fernández-Martínez, Andrea Barros-Martínez, María Cristina Martínez-Fernández, Marta Quiñones-Pérez

**Affiliations:** 1 SALBIS Research Group, Faculty of Health Sciences, University of León, 24071 León, Spain; 2 Emergency Department, Complejo Asistencial Universitario de León CAULE, 24008 León, Spain; 3 SALBIS Research Group, Faculty of Health Sciences, Ponferrada Campus, University of León, Ponferrada, 24401 León, Spain

**Keywords:** suicide attempt, emergencies, COVID-19

## Abstract

Suicide is a significant public health concern, with one million lives lost to it every year. Suicidal ideation and attempts are markers of high risk. The COVID-19 pandemic has had a negative psychological impact on the population. This study aims to describe and analyze the clinical and sociodemographic characteristics of patients who have received medical attention for self-harm attempts in a hospital emergency department, comparing the period before and after the COVID-19 pandemic. This is a descriptive, retrospective study that collected data from medical records of patients who received care for self-harm attempts in the emergency department. The data included cases from 1 January 2018 to 31 December 2022. In total, 529 cases of self-harm attempts were identified, of which 62.8% were female. The number of post-pandemic self-harm attempts significantly increased compared to the period before the pandemic. The most used method for self-harm was medication ingestion. This study revealed that over one-third of the participants had previously attempted suicide. Most self-harm attempts were made by women in the 10–20 or 41–50 age groups, with a history of psychiatric illness and multiple medications. The study results also highlighted an increase in self-harm attempts during the COVID-19 pandemic.

## 1. Introduction

The World Health Organization (WHO) states that suicide claims approximately one million lives globally each year [1], translating to about 3000 individuals daily. In Spain, suicide-related deaths saw a 1.6% increase from 2020, with a total of 4003 fatalities reported in 2021 [2]. Acts of self-harm, defined as intentional self-inflicted harm with potential suicidal outcomes, are a significant concern. The National Statistics Institute of Spain (INE) approximates that, for every suicide, there are between ten and forty suicide attempts [3,4]. Both suicidal ideation and intent are considered high-risk indicators, and addressing these factors is vital in averting progression to more severe stages. However, the WHO does not officially report data on this subject [5,6].

Self-harm is a significant risk factor for suicide mortality [7]. Therefore, it is crucial to acknowledge that there are several overlooked stages preceding suicide within the scope of suicidal behavior. These stages, as mentioned in the text, include periods of help-seeking and may signal underlying mental health issues. Some researchers propose that if these risks can be identified and mitigated, suicide could be categorized as a preventable health concern [5,8,9]. Studies have shown that the risk of suicide escalates in the six months following a self-harm attempt, with the initial month being the most critical. Consequently, it is imperative to provide post-discharge follow-up care to avert future attempts [10,11]. This underscores the necessity to highlight the significance of prevention and promotion, a requirement for programs that educate about emotional health, and the availability of therapeutic tools that can deter short-term suicide attempts and prevent the loss of numerous lives in the long term [5]. Therefore, understanding the characteristics that predispose individuals to self-harm attempts, identifying the most susceptible groups, and directly targeting them is required.

A positive correlation exists between social isolation and suicide, with social support being deemed a protective factor [12]. Suicidal actions are often triggered in crisis situations, such as financial hardships, familial disputes, chronic diseases, romantic separations, bereavement, or instances of abuse [11]. Contrary to expectations, a study has shown that educational attainment is not an effective means of equipping individuals with life skills [13]. A study conducted in Spain suggests that the most common triggers for suicidal behavior are related to problems within couples and families. In both cases, arguments are a frequent factor, with women being more likely to exhibit suicidal behavior [14]. The WHO has formulated the ‘LIVE LIFE’ Guide to counter suicide through measures like the early detection of suicidal thoughts or behaviors, monitoring individuals at risk, and imparting socioemotional skill education from adolescence [15].

The COVID-19 pandemic resulted in the cessation of public activities and the implementation of restrictive measures. In Spain, the pandemic brought about by the COVID-19 virus forced the government to declare a state of alarm (Royal Decree 463/2020, of 14 March), for the management of the health crisis situation [16]. Strict control measures were implemented in Spain to combat the COVID-19 outbreak. Initially, travel within the country’s regions was prohibited, and people were only allowed to leave their homes for essential activities such as shopping or accessing health services. Later, time slots were established for people to access the streets, and de-escalation began by limiting crowded spaces [17]. The activation of these health emergency measures helped to bring the COVID-19 outbreak in Spain under control, and the de-escalation phases began in May. The state of alert ended on 21 June 2020 [18]. Several studies have confirmed that this has had a negative psychological impact on the population, which can be attributed to the loss of routine and a significant reduction in physical and social contact [10,19,20,21,22]. Furthermore, the economic impact of the pandemic, including increased unemployment, may lead to a rise in suicide attempts among individuals without a history of mental illness [23]. Moreover, adolescents are a particularly vulnerable group due to their developmental stage and their need to satisfy their peers, making them susceptible to future psychological adjustment issues. Changes in society and its problems can have an impact on young people’s attitudes and values. It is important to reinforce positive behaviors to help children build a positive self-image and encourage their participation in decision-making. This strengthens their role in society. The proper management of free time is crucial for their health [24]. During quarantine, individuals may experience isolation and loneliness, which can increase the risk of suicide [5,19,21,25,26]. The European Health Survey in Spain reported an increase in disinterest in activities outside of everyday life or feelings of depression or sadness during the pandemic [27]. The WHO highlights the significant issue of mental health disorders affecting almost one billion people, resulting in a negative impact on their quality of life. A systematic review of the influence of COVID-19 on suicide attempts indicates that the unforeseen behavioral changes during the pandemic may have played a role in the escalating trend of suicide attempts [28].

The pandemic has been recognized as a substantial factor leading to emotional instability in the aftermath of confinement. Research has indicated a 25% surge in instances of depression and anxiety during the pandemic’s initial year [1]. However, there is a paucity of research investigating whether this increase has precipitated a rise in suicide attempts [5,11,19,29]. Consequently, this study’s primary objective is to delineate and contrast cases of attempted suicide and their attributes pre- and post-COVID-19 pandemic within a tertiary hospital’s emergency department.

## 2. Materials and Methods

### 2.1. Design and Sample

A retrospective, descriptive study was undertaken, reviewing the medical records of patients who necessitated intervention from the Mental Health Service due to a self-harm attempt (including suicide attempts) when presenting at the emergency department of a tertiary hospital in the community of Castile and León.

Data were collected for the period before and after the COVID-19 pandemic, from 1 January 2018 to 31 December 2022. The resultant sample was bifurcated into two cohorts: the pre-pandemic period, encompassing the years 2018 and 2019, and the post-pandemic period, which included the years 2021 and 2022.

### 2.2. Procedure

All medical records encompassed diagnoses pertinent to self-harm attempts, which included suicide attempts, self-harm, or self-harming gestures. In the hospital data, the terms ‘suicide attempts’ and ‘self-harm gestures’ were used interchangeably. However, for the purposes of this study, we defined them collectively as self-harm attempts. These attempts spanned a broad spectrum of methods, such as drug or alcohol injection or ingestion, chemical ingestion, hanging, drowning, falling from a height (precipitation), weapon use, electrocution, gas inhalation, burns, blows, or an amalgamation of several of these methods. Exclusions were instituted for instances of alcohol and/or drug intoxications that were recreational, unintentional, or accidental.

### 2.3. Variables

Both quantitative and qualitative variables were included. The quantitative variables comprised age, the number of previous psychiatric admissions, and the number of previous self-harm attempts. The qualitative variables encompassed gender, marital status, reason for consultation, psychiatric history, medication, the intentionality of the act, the method employed, and the patient’s destination following medical care.

### 2.4. Data Analysis

The data were scrutinized by utilizing the Statistical Package for the Social Sciences (SPSS) software, version 27.0. Quantitative variables were delineated as the mean and standard deviation, while qualitative variables were expressed as frequencies and percentages. The correlation between variables was analyzed employing Student’s *t*-tests, chi-square tests, and Spearman’s correlations. The threshold for statistical significance was established at *p* < 0.05.

### 2.5. Ethical Considerations

The anonymity and confidentiality of the subjects throughout the research process were ensured. The research received approval from the ethics committee of the health area to which the hospital belongs (protocol code: 2337), as well as permission from the hospital management. The data were processed in accordance with the Organic Law on Data Protection 7/2021 and the recommendations of the Helsinki Declaration of 1975, revised in 2008.

## 3. Results

### 3.1. Sociodemographic Characteristics

From 2018 to 2022, a total of 529 patients were admitted to the emergency department due to self-harm attempts. Of these, 36.3% (n = 192) were male, 62.8% (n = 332) were female, and 0.9% (n = 5) identified as non-binary. The mean age of the individuals was 42.74 (±18.88; max. 96, min. 11). The analysis of the age groups revealed that the largest proportion of individuals fell within the 41–50 age range (n = 130; 24.6%). Table 1 presents the descriptive data for the pre- and post-pandemic periods, as well as the association between them. In 2018, a total of 101 patients with a self-harm attempt diagnosis attended the emergency department. This number decreased to 84 in 2019, and in 2020, 82 cases were recorded. In both 2021 and 2022, 131 individuals attended each year. Statistically significant differences were observed in the number of cases between the two periods, with an increase in the post-pandemic period (t (446) = 68.01, *p* = 0.0001). Table 2 presents the correlation analyses between age, previous self-harm attempts, and previous admissions to psychiatry. The analysis revealed a negative and significant correlation between age and self-harm attempts. Research has shown a negative correlation between age and self-harm attempts, while previous self-harm attempts were positively correlated with previous admissions to psychiatric units (Table 1).

### 3.2. Previous Psychiatric History and Treatment

Table 3 presents the psychiatric history and treatment profiles of the patients. In the post-pandemic period, there was a significant increase in the correlation between self-harm attempts and a history of mental health problems (χ2(19) = 38.315, *p* = 0.005), particularly anxiety and depression (increased from 13.5% to 16.8%) and in patients diagnosed with multiple psychiatric conditions (increased from 15.1% to 27.5%). In terms of treatment, the post-pandemic period saw a notable increase in the number of patients prescribed psychiatric medication (χ2(8) = 15.419, *p* = 0.031). Furthermore, there was a growth in the percentage of patients on multiple medications (from 49.2% to 58.4%) and in the use of anxiolytics (from 8.1% to 12.2%).

### 3.3. Characteristics of Self-Harm Attempts

In terms of the intentionality of the act, 78.8% (n = 417) of individuals expressed a desire to end their own lives, while 11.2% (n = 59) refuted any such intention, and 10% (n = 53) were uncertain about their intent. Table 4 outlines the specifics of the self-harm attempts. The most commonly used method was drug ingestion, followed by sharp force trauma. The majority of the patients were able to return home. It is important to note that 63.5% of the cases had not attempted autolysis previously, and almost all (90.7%) had not been admitted to psychiatric care before. The fatality rate was only 0.6%. Notably, there were no statistically significant differences in the characteristics associated with self-harm attempts between the pre-pandemic and post-pandemic periods.

## 4. Discussion

Presently, there are concerns about estimating the impact of the COVID-19 pandemic on the mental health of the population [10,11,22]. The COVID-19 pandemic has had a severe impact on the mental health of the population. Social isolation and disruptions in health services may have contributed to an increased risk of suicidal ideation during the pandemic, leading to higher rates of suicide and mental illness [30]. This study aimed to address this issue by describing and comparing the instances and characteristics of self-harm attempts before and after the COVID-19 pandemic in an emergency department. The findings suggest a significant rise in the number of self-harm attempts in the post-pandemic period compared to the pre-pandemic period. These results are in contrast with another study that reported a decrease in the number of self-harm attempts in an emergency department post-pandemic, with most of the individuals being single and older [29]. Mongodi et al. reported an increase in the number of admissions to ICUs due to self-harm post-pandemic [31]. Another study reported a 38.5% increase in suicide attempts post-pandemic, but this increase was not statistically significant due to the short duration of the analysis period [32]. It is crucial to consider factors such as conflict, domestic violence, economic loss, anxiety and depression, or pre-existing mental disorders to prevent suicide attempts [28]. Yan et al. (2021) carried out a meta-analysis and discovered a significant surge in suicide ideation and attempts during the COVID-19 pandemic. However, the rates of suicide deaths have remained stable [33]; these results align with our findings. Moreover, individuals who were infected with COVID-19 reported an increase in suicidal thoughts and risk behaviors [34]. In the context of the pandemic, dissatisfaction with close relationships and housing emerged as the most significant predictors of suicidal ideation, surpassing the economic situation. Machine learning techniques can be employed to identify the presence of suicidal ideation [35].

In prior research, a gender disparity has been observed in the frequency of self-harm attempts, with women reporting a higher number of attempts than men [5,6,9,22,25,26,36]. No correlation was identified between relationship status and drug use across the two periods under study, although such a relationship is evident in the context of psychiatric history and treatment. Current evidence suggests that confinement in isolation and uncertainty can have negative consequences, leading to an increase in mental health problems such as anxiety and depression [9,11,19,20,25]. In contrast, a study conducted in the Czech Republic found that the happiness and quality of life of individuals during the peak of the pandemic was higher than that measured one year before the pandemic. This could be due to the fact that they were in an adequate health state during a period of uncertainty and global health emergency [37]. In this study, we observed an increase in both pathologies in post-pandemic patients, as well as an increase in the number of people with multiple pathologies.

In line with previous studies, there was an increase in the number of individuals on multiple medications post-confinement, accompanied by an increased consumption of anxiolytics [5,10,22]. Drug ingestion emerged as the predominant method of self-harm, consistent with other studies [38]. Of the total self-harm attempts, 0.6% resulted in fatalities due to complications. One potential repercussion of COVID-19 is the detrimental impact it can have on individuals who have lost a loved one, potentially leading to heightened suicidal ideation, complicated grief, and depression. It is noteworthy that these effects have been documented in studies such as the one referenced in [39]. Although recent official data at the Spanish level are lacking, a study conducted in Spain suggests that consultations for suicidal ideation and attempts have increased during the COVID-19 pandemic compared to the previous two years [40]. Additionally, there has been an increase in suicidal ideation and attempts following home confinement [40], which is consistent with our results.

Finally, it is worth noting that the age range with the greatest increase in self-harm attempts after the pandemic was 41 to 50 years. These data are comparable to those reported by Kim et al. In their study, the highest number of self-harm attempts occurred in the 40–49 age group, followed by the 50–59 age group, which had the highest percentage of suicide deaths [41]. However, a study conducted in India indicates that the highest number of self-harm attempts occur among women and individuals aged 18–29 years old [42]. Similar to another study conducted in an emergency department in Ethiopia, the most frequent age range was found to be between 28 and 37 [43]. However, a study conducted in Taiwan suggests an increased risk of suicide in vulnerable groups, including those under 25 and over 65 years of age [44]. There has also been a significant rise in attempts among adolescents aged 10 to 20 years. This evidence underscores the vulnerability of adolescents during confinement and exclusionary situations, as well as the psychological adaptation challenges they face during these times [11,19,26]. A study conducted in the United States revealed that in the months following the pandemic, there was a substantial increase in calls to poison control centers due to suicidal intentions in adolescents, resulting in a 44% increase in fatalities [45]. Another study carried out in Korea reported a 2.2% increase in suicide attempts among adolescents. This was partly related to economic stress and feelings of loneliness [46]. Therefore, it is important to understand the level of life satisfaction among young people and the factors that can impact their well-being [47].

This study has limitations. Although the sample size is considerable, it only includes individuals seeking care in the emergency department, which does not represent the entire population of those who may have attempted self-harm. Additionally, the results cannot be generalized due to the geographical limitation. Furthermore, the retrospective approach of this study prevents obtaining detailed information on the underlying motives that led the subjects to attempt suicide. It is important to note that we were unable to follow up with patients after their stay in the emergency department.

## 5. Conclusions

Three years after the COVID-19 lockdown, it is evident that the pandemic has had a significant impact on the mental health of the population, both directly and indirectly. The increase in self-harm attempts following the pandemic highlights the need for prevention plans and patient follow-up in cases of psychological and emotional vulnerability.

Risk factors for self-harm attempts have been observed in individuals with certain characteristics. These include female gender, 10–20 and 41–50 age groups, a history of psychiatric disorders (particularly multipathological disorders such as anxiety and depression), being on multiple medications (especially antidepressants and anxiolytics), and previous admissions to psychiatric units. The number of attempted suicide cases increased by 14.5% in the post-pandemic stage compared to the pre-pandemic period.

Therefore, it is crucial to monitor and identify high-risk patients in emergency departments. It is also essential to assess the impact of the pandemic on mental health and implement prevention and early detection programs for at-risk situations.

## Figures and Tables

**Table 1:** Sociodemographic characteristics of the sample.

	Total Sample	Pre-Pandemic (2018–2019)	Post-Pandemic (2021–2022)	*p*
	n (%)	n (%)	n (%)	
Gender				0.620
Male	192 (36.3%)	66 (35.7%)	92 (35.1%)	
Female	332 (62.8%)	118 (63.8%)	166 (63.4%)	
Non-binary	5 (9%)	1 (0.5%)	4 (1.5%)	
Marital Status				0.963
Unknown	225 (42.5%)	83 (44.9%)	113 (43.1%)	
Single	40 (7.6%)	12 (6.5%)	20 (7.6%)	
Couple	84 (15.9%)	30 (16.2%)	41 (15.6%)	
Married	78 (14.7%)	25 (13.5%)	40 (15.3%)	
Separated	85 (16.1%)	31 (16.8%)	40 (15.3%)	
Widowed	17 (3.2%)	4 (2.2%)	8 (3.1%)	
Prior Drug Use				0.128
No	404 (76.4%)	134 (72.4%)	206 (78.6%)	
Yes	116 (21.9%)	49 (26.5%)	50 (19.1%)	
Ex-consumer	9 (1.7%)	2 (1.1%)	6 (2.3%)	

**Table 2:** Correlations between age, psychiatric admissions, and previous self-harm attempts.

		Age	Previous Self-Harm Attempts
Previous self-harm attempts	Correlation coefficient	−0.100 *	
	Sig. (bilateral)	0.034	
Previous admissions to psychiatry	Correlation coefficient	−0.034	0.329 **
	Sig. (bilateral)	0.478	0.000

* *p* < 0.05, ** *p* < 0.01.

**Table 3:** Association between history and previous psychiatric treatment.

	Total Sample	Pre-Pandemic (2018–2019)	Post-Pandemic (2021–2022)	*p*
	n (%)	n (%)	n (%)	
Psychiatric history				0.005 **
No	128 (24.2%)	49 (26.5%)	56 (21.4%)	
Anxiety	41 (7.8%)	14 (7.6%)	18 (6.9%)	
Depression	73 (13.8%)	31 (16.8%)	31 (12.2%)	
Anxiety and depression	85 (16.1%)	25 (13.5%)	44 (16.8%)	
Gender dysphoria	3 (0.6%)	1 (0.5%)	2 (0.8%)	
BPD	26 (4.9%)	15 (8.1%)	9 (3.5%)	
ADHD	5 (0.9%)	0 (0%)	4 (1.5%)	
Adaptive/dissociative identity disorder	24 (4.5%)	10 (5.4%)	13 (5%)	
Schizophrenia/schizoaffective disorder	4 (0.8%)	0 (0%)	2 (0.8%)	
OCD	1 (0.2%)	0 (0%)	1 (0.4%)	
Emotional instability/dysthymia/reactive attachment disorder	5 (1%)	1 (0.5%)	2 (0.8%)	
Bipolar disorder	7 (1.3%)	5 (2.7%)	1 (0.4%)	
Epilepsy	5 (0.9%)	2 (1.1%)	3 (1.1%)	
Psychotic disorder	5 (0.9%)	4 (2.2%)	1 (0.4%)	
Eating Disorder	2 (0.4%)	0 (0%)	2 (0.8%)	
Pluripathological (>2)	115 (21.7%)	28 (15.1%)	72 (27.5%)	
Psychiatric treatment				0.031 **
No	133 (25.1%)	63 (34.1%)	51 (19.5%)	
Polymedication (>2)	292 (55.2%)	91 (49.2%)	153 (58.4%)	
Antidepressant	37 (7%)	14 (7.6%)	20 (7.6%)	
Anxiolytic	58 (10.9%)	15 (8.1%)	32 (12.2%)	
Antipsychotic	2 (0.4%)	1 (0.5%)	1 (0.4%)	
Anticonvulsant	1 (0.2%)	0 (0%)	0 (0%)	
Treatment for ADHD	2 (0.4%)	0 (0%)	2 (0.8%)	
Hypnotic	2 (0.4%)	0 (0%)	2 (0.8%)	
Antiepileptic	2 (0.4%)	1 (0.5%)	2 (0.8%)	

ADHD: attention-deficit hyperactivity disorder, BPD: borderline personality disorder, OCD: obsessive–compulsive disorder. ** *p* < 0.01

**Table 4:** Characteristics of self-harm attempts.

	Total Sample	Pre-Pandemic (2018–2019)	Post-Pandemic (2021–2022)	*p*
	n (%)	n (%)	n (%)	
Intent of the act				0.474
Unknown	53 (10%)	21 (11.4%)	25 (9.5%)	
Death	417 (78.8%)	141 (76.2%)	212 (80.9%)	
Denies self-intention	59 (11.2%)	23 (12.4%)	25 (9.5%)	
Method of self-harm				0.149
Various methods	36 (6.8%)	9 (4.9%)	24 (9.2%)	
Drug ingestion	340 (64.3%)	112 (60.5%)	174 (66.4%)	
Drug injection	4 (0.8%)	1 (0.5%)	2 (0.8%)	
Chemical ingestion	11 (2.1%)	5 (2.7%)	6 (2.3%)	
Precipitation	26 (4.9%)	13 (7%)	8 (3.1%)	
Drowning	2 (0.4%)	2 (1.1%)	0 (0%)	
Hanging	19 (3.6%)	9 (4.9%)	6 (2.3%)	
Sharp force trauma	73 (13.8%)	27 (14.6%)	33 (12.6%)	
Firearm	2 (0.4%)	0 (0%)	2 (0.8%)	
Self-harm ideas	6 (1.1%)	2 (1.1%)	3 (1.1%)	
Electrocution	1 (0.2%)	0 (0%)	1 (0.4%)	
Gas inhalation	7 (1.3%)	4 (2.2%)	2 (0.8%)	
Burns	1 (0.2%)	1 (0.5%)	0 (0%)	
Beatings	1 (0.2%)	0 (0%)	1 (0.4%)	
Patient destination				0.478
Admission to psychiatry	139 (26.3%)	49 (26.5%)	66 (25.2%)	
Discharge to home	353 (66.7%)	123 (66.5%)	176 (67.2%)	
Voluntary discharge	25 (4.7%)	9 (4.9%)	14 (5.3%)	
ICU	8 (1.5%)	1 (0.5%)	5 (1.9%)	
Runaway	4 (0.8%)	3 (1.6%)	1 (0.4)	
Previous self-harm attempts				
No	336 (63.5%)	124 (67%)	162 (61.8%)	
1	110 (20.8%)	34 (18.4%)	52 (19.8%)	
2	46 (8.7%)	13 (7%)	27 (10.3%)	
3	28 (5.3%)	12 (6.5%)	14 (5.3%)	
4	7 (1.3%)	2 (1.1%)	5 (1.9%)	
5	1 (0.2%)	0 (0%)	1 (0.4%)	
6	1 (0.2%)	0 (0%)	1 (0.4%)	
Previous psychiatric admissions				0.811
No	480 (90.7%)	169 (91.4%)	241 (92%)	
Yes	49 (9.3%)	16 (8.6%)	21 (8%)	
Deceased after the self-harm attempt				0.811
No	526 (99.4%)	184 (99.5%)	260 (99.2%)	
Yes	3 (0.6%)	1 (0.5%)	2 (0.8%)	

## Data Availability

Data are contained within the article.
